# The Roads to Mitochondrial Dysfunction in a Rat Model of Posttraumatic Syringomyelia

**DOI:** 10.1155/2015/831490

**Published:** 2015-01-13

**Authors:** Zhiqiang Hu, Jian Tu

**Affiliations:** ^1^Department of Neurosurgery, Beijing Shijitan Hospital, Capital Medical University, Beijing 100038, China; ^2^Prince of Wales Clinical School, The University of New South Wales, Sydney, NSW 2052, Australia

## Abstract

The pathophysiology of posttraumatic syringomyelia is incompletely understood. We examined whether local ischemia occurs after spinal cord injury. If so, whether it causes neuronal mitochondrial dysfunction and depletion, and subsequent energy metabolism impairment results in cell starvation of energy and even cell death, contributing to the enlargement of the cavity. Local blood flow was measured in a rat model of posttraumatic syringomyelia that had received injections of quisqualic acid and kaolin. We found an 86 ± 11% reduction of local blood flow at C8 where a cyst formed at 6 weeks after syrinx induction procedure (*P* < 0.05), and no difference in blood flow rate between the laminectomy and intact controls. Electron microscopy confirmed irreversible neuronal mitochondrion depletion surrounding the cyst, but recoverable mitochondrial loses in laminectomy rats. Profound energy loss quantified in the spinal cord of syrinx animals, and less ATP and ADP decline observed in laminectomy rats. Our findings demonstrate that an excitotoxic injury induces local ischemia in the spinal cord and results in neuronal mitochondrial depletion, and profound ATP loss, contributing to syrinx enlargement. Ischemia did not occur following laminectomy induced trauma in which mitochondrial loss and decline in ATP were reversible. This confirms excitotoxic injury contributing to the pathology of posttraumatic syringomyelia.

## 1. Introduction

Following spinal cord injury, posttraumatic syringomyelia or cystic degeneration of the spinal cord develops in up to 30% of patients [1–4]. Most of these patients do not respond well to the current therapeutic options because the underlying mechanisms causing the formation and enlargement of such cavities are not fully understood [5]. Spinal cord trauma triggers several pathophysiological events, including a massive release of glutamate, hyperactivation of *N*-methyl-D-aspartate receptors (NMDARs), and ischemia, which contribute to neuronal death and syrinx formation. An acute release of glutamate at the site of injury occurs within hours and days of spinal cord trauma [6–8]. Glutamate mediates excitatory synaptic transmission through the activation of ionotropic glutamate receptors that are sensitive to *N*-methyl-D-aspartate (NMDA), *α*-amino-3-hydroxy-5-methyl-4-isoxazolepropionic acid (AMPA), or kainite, promoting arachnoiditis.

Local ischemia may be in part due to obliteration of spinal cord vessels. Ischemia is defined as impaired blood supply to a tissue or organ leading to inadequate oxygen delivery. If the ischemic process proceeds, hypoxia progresses to anoxia for the cell component, because of the selectively high affinity of mitochondrial cytochrome oxidase for oxygen. Early structural changes due to hypoxia include mitochondrial rounding and moderate cell swelling. These changes are reversible if reoxygenation occurs. Late in hypoxic stress, however, a distinct intermediate state develops that is characterized by mitochondrial permeabilization, leakage of low-molecular-weight anionic solutes across the plasma membrane and accelerates cell swelling. Rapid swelling during the intermediate state is driven by the oncotic (colloid osmotic) pressure of macromolecules in the cytosol. Continued mitochondria swelling leads to membrane stretching and ultimately bursting of the plasma membrane. Rupture of plasma membrane brings about irreversible cellular injury and onset of necrotic cell death.

Ischemia triggers a sequence of events that lead to neuronal cell necrosis or apoptosis. The degree of ischemia may relate to the relative amounts of necrosis or apoptosis. Although necrosis and apoptosis have long been considered totally distinct phenomena, apoptotic and necrotic features are, in fact, often coexisting [9], which can occur parallel in neuronal cells after ischemia [9]. Cell death processes may begin with common signals and stresses, progress through shared pathways, such as mitochondrial permeabilization, and culminate in either cell lysis (necrosis) or programmed cellular resorption (apoptosis), the term necrapoptosis has been introduced [9]. In necrapoptosis, pure apoptosis and pure necrosis are extremes in a spectrum of changes in response to stresses and death signals, but the more common pathophysiological response is a mixture of features associated with both apoptotic and necrotic cell death. Often, the intensity of ischemia and the status of cellular energy levels decide which process is more prevalent, and mitochondria, by controlling ATP production, seem to act as a switch between a more prominent necrotic or the apoptotic demise. For example, a subacute ischemia may leave affected neurons with injured but still partially functioning mitochondria, therefore, generating sufficient ATP levels to allow apoptosis to fully develop. Fulminant ischemia, by contrast, results in the rapid compromise of mitochondrial function and subsequently intracellular energy levels, leaving cells no other option than to abort the apoptotic program and switch to necrosis.

Mitochondria are uniquely poised to play a pivotal role in neuronal cell survival or death after ischemia because they are regulators of both energy metabolism and apoptotic pathways. Maintaining mitochondrial homeostasis and bioenergetics in neurons is even more critical because of their almost complete dependence on mitochondrial-derived ATP [10].

An interrupted blood supply to neuronal tissue causes deprivation of oxygen and nutrients which are utilized to produce ATP in neuronal mitochondria. Impaired ATP production increases presynaptic glutamate release through membrane depolarization and the subsequent activation of the voltage-gated Ca^2+^ channel. It also interferes with the reuptake of glutamate into astrocytes, which results in the abnormal accumulation of synaptic glutamate. Excess and sustained activation of the ionotropic glutamate receptors causes rapid, irreversible ATP loss resulting in fulminant spinal neuronal death [11], namely, glutamate neurotoxicity. In our animal model microinjection of quisqualic acid, an agonist of ionotropic and group I metabotropic glutamate receptors, into the spinal cord grey matter produces similar glutamate neurotoxicity of human posttraumatic syringomyelia [12].

It has been suggested that excitotoxic insult causes severe damage to neuron cell bodies [13]. Neuronal loss has been documented at the time of spinal cord injury and the time of syrinx formation as well as the time of syrinx enlargement [12]. This cell loss may be directly resultant from ischemia; alternatively, ischemia can serve as a secondary factor caused by increasing cerebrospinal fluid (CSF) pressure against the syrinx wall. There is supporting evidence from clinical studies that syrinx decompression immediately increases local blood flow and improves motor neuron electrical conduction and neurological function [14–16]. We hypothesize that the combination of excitotoxic insult and kaolin-induced arachnoiditis is associated with mitochondrial dysfunction and depletion, poor energy metabolism, and subsequent neuronal loss, which contributes to syrinx enlargement. The aims of this study were to investigate the combining effects of excitotoxic insult and kaolin-induced arachnoiditis on neuronal mitochondria and consequences.

## 2. Materials and Methods

Animal experimentation was approved and performed in accordance with the guidelines of the institutional Experimental Animal Care and Ethics Committee, Guide for the Care and Use of Laboratory Animals (Institute for Laboratory Animal Research, National Research Council. Washington, D.C.: National Academy Press, 1996), and the Code of Practice for the Care and Use of Animals for Scientific Purposes [17]. Seventy-two 10-week-old male inbred Wistar rats (355 ± 8 g) were randomly divided into normal controls (intact, *n* = 24) and sham-operated (laminectomy, *n* = 24) control groups as well as an experimental syrinx group (syrinx, *n* = 24) receiving a combination of intraparenchymal quisqualic acid and subarachnoid kaolin for the study. Four animals were studied at each of three survival time points (1 hour, 24 hours, and 6 weeks) for the quantification of spinal cord blood flow and adenine nucleotides; another 4 rats per time point for examining ultrastructural changes in neuronal mitochondria and histology.

### 2.1. Syrinx Formation

The syrinx induction procedure has been previously described in details [12, 18, 19]. Briefly, the dura and arachnoid were opened following a C7 to T1 laminectomy. A glass needle tipped (outside diameter 50 *μ*m) 5 *μ*L syringe (SGE, Australia) held in a stereotactic micromanipulator was used to infiltrate four 0.5 *μ*L injections of 24 mg/mL quisqualic acid (Tocris Cookson Ltd., Bristol, UK) and 1% Evans blue at separate points along the right dorsal nerve rootlets from the rostral C8 to the caudal T1 levels. Quisqualic acid was dissolved in four-fifths 0.9% saline and one-fifth 1% Evans blue which allowed any leakage of the quisqualic acid to be identified. To promote a dense adhesive arachnoiditis, 10 *μ*L of 250 mg/mL kaolin (Sigma-Aldrich, St Louis, MO, USA) in 0.9% saline were injected into the subarachnoid space. This model has previously been shown to result in expanding syrinxes in the majority of experimental animals [12, 18, 19]. Rats in the intact group had no surgery, the sham operated group had laminectomies only. At survival time points, spinal cord blood flow was measured before spinal cord segment at C8 was harvested for electron microscopy and histology or immediately placed in liquid nitrogen. Specimens remained in liquid nitrogen until measurement of adenosine nucleotides. Tissue of laminectomy and intact controls was processed identically.

### 2.2. Spinal Cord Blood Flow Measurement

Spinal cord blood flow measurement has been performed. Briefly, the dura and arachnoid were opened following a C8 to T1 laminectomy. Spinal cord perfusion was measured using an optical laser Doppler probe (Model MP4s, G26 tube with a blunt end diameter 0.46 mm: Moor Instruments, Axminster, Devon, UK). The MP4s probe was connected to a laser Doppler blood flow imaging system (moorLDI2: Moor Instruments) and data recorded through a data acquisition module (PowerLab 16/35: AD Instruments, Castle Hill, NSW, Australia).

Spinal cord blood flow was measured following the intradural procedure at C8 level at 1 hour, 24 hours, and 6 weeks after syrinx induction. Blood flow was relative to the initial reading in intact control rats, which was expressed as a percentage change in baseline values, adjusted to 100.

### 2.3. Histology

Animal perfusion, tissue processing, and hematoxylin and eosin (H and E) staining have been previously described [12, 18, 19]. Briefly, 7 *μ*m paraffin cross-sections of spinal cord were dewaxed with xylene and an alcohol series. Slides were placed in hematoxylin for 5 minutes, followed by a dip in 2% acid alcohol and then in lithium carbonate. The slides were dipped in eosin for 2 minutes and then dehydrated with ethanol before xylene. H and E stained sections at C8 level were divided into 838 ± 32 grid squares for semiquantification of cyst size. Syrinxes were observed as cystic spaces within the cord, sometimes containing cellular debris and a loose trabecular meshwork. The syrinx margin was defined as an interface between normal tissue and cystic spaces. Syrinx size was measured as a percentage of cross-sectional area of the entire section (number of grid squares containing ≥50% cyst/number of grid squares per complete spinal cord section).

### 2.4. Electron Microscopy and Analysis

Specimen processing for ultrastructural study has been previously described in detail [20–22]. Briefly, 1 mm^3^ fragments were randomly taken from grey matter at C8 level and fixed in 2% paraformaldehyde and 1% glutaraldehyde in 0.1 mol/L sodium cacodylate at 4°C for 2 hours. The specimens were postfixed in 1% OsO_4_ at 25°C for 1 hour. After rinsing in 0.1 mol/L sodium cacodylate, the specimens were initially stained* en bloc* with 2% uranyl acetate for 1 hour and sequentially dehydrated in increasing concentrations of ethanol (EtOH) (50%, 70%, 90% and 100% EtOH 10 min, resp.; absolute dry EtOH 30 min; a 1 : 1 dry EtOH-propylene oxide mixture 15 min; and propylene oxide 15 min). Before embedding, tissue was sequentially infiltrated by a serial immersion in increasing concentrations of epoxy (a mixture of 1 part propylene oxide : 1 part epoxy and 1 : 3 propylene oxide-epoxy for 1 hour, resp., and pure epoxy resin overnight). The specimens were embedded in pure epoxy resin and polymerized at 70°C for 72 hours. Laminectomy and intact control specimens were sampled and processed identically.

Neuronal tissue was identified by light microscopy on semithin sections stained with 1% toluidine blue. Multiple, serial ultrathin sections of neuronal tissue were cut with an ultratome, and 50 nm sections were collected on grids coated with polyvinyl formal support film. The sections were stained with Reynold's lead citrate solution to enhance contrast. Transmission electron microscopy was used to evaluate and analyze neuronal mitochondria. Ultrastructural findings and density of mitochondria per neuron on electron micrographs were examined by three observers blinded to the sample nature.

### 2.5. Adenine Nucleotide Assay

ATP, ADP, and AMP were quantified in triplicate by a luminometric method [23]. For ATP measurement, a commercial ATP bioluminescence assay kit CLS II (Roche Molecular Biochemicals, Mannheim, Germany) was used. Each sample was homogenised on ice, then suspended in 100 *μ*L of a boiling solution containing 100 mmol/L Tris and 4 mmol/L EDTA (pH 7.75), and incubated at 100°C for 2 min followed by centrifugation at 10,000 ×g for 60 sec at 4°C. One hundred microliters of cellular extract supernatant were collected and subsequently placed in a luminometer (TD-20/20, Turner Designs, Promega, U.S.A.). Light emission was quantified after addition of 100 *μ*L of a commercial ATP monitoring reagent containing firefly luciferase.

For ADP measurement, cellular extract supernatant from another portion of each sample was incubated for 45 min at 25°C with a rephosphorylation buffer containing 1.5 mmol/L phosphoenolpyruvate and 2.3 U/mL pyruvate kinase to convert ADP into ATP. For AMP measurement, cellular extract supernatant from a portion of each sample was incubated for 45 min at 25°C with a rephosphorylation buffer containing 1.5 mmol/L phosphoenolpyruvate, and 36 U/mL adenylate kinase to convert AMP into ADP prior to a second incubation with 2.3 U/mL pyruvate kinase for 45 min at 25°C to convert ADP to ATP. ATP was then measured as described above, and ADP and AMP levels were calculated by the difference. Reagent blanks, ATP, ADP, and AMP standards were used through the entire procedure.

The concentration of protein in each sample was quantified using a commercial protein assay kit (Bio-Rad Protein Assay; Bio-Rad Laboratories Inc., Hercules, CA, U.S.A.). Briefly, a protein sample was mixed with a dye reagent producing a colour change that was measured with a microplate reader (iMark; Bio-Rad Laboratories Inc.), and compared to a standard curve of known protein concentrations.

Data were expressed as ATP, ADP, or AMP nmol/mg protein.

### 2.6. Data Analysis

Data were expressed as the mean ± SE (number of experiments). Statistical difference between groups was determined by the unpaired two-tailed Student's *t* test. When there were more than two groups, differences were analyzed using analysis of variance if the variances were equal, and the Mann-Whitney nonparametric test if variances were unequal [24]. A value of *P* < 0.05 was considered significant. Software used included Excel (Microsoft Corp., Redmond, Washington, U.S.A.) and SPSS (Statistical Package for the Social Sciences, SPSS Inc., Chicago, Illinois, U.S.A.).

## 3. Results

### 3.1. Syrinx Formation

Comparing to normal control and sham-operated rats, all rats developed a syrinx by 6 weeks after receiving combined intraparenchymal quisqualic acid and subarachnoid kaolin injection. Syrinx occurred in the grey matter and at the level of quisqualic acid injection. Syringes ranged from 1 to 3 segments in length and the average size was 14.7 ± 0.6% of the cross-sectional area at the C8 level. Most of the syringes were unilocular but irregular in shape. No syrinx formed in all groups at 1 hour and 1 day time points. No syrinx formed in normal control and sham-operated rats.

### 3.2. Reduction of Spinal Cord Blood Flow

Local blood flow at C8 revealed 85.7 ± 11.4% reduction in syrinx animals when compared to laminectomy at 6 weeks after syrinx induction procedure (*P* < 0.05). There were no significant differences in local blood flow rate in the spinal cord between the laminectomy and intact controls at 6-week time point. There were also no significant alterations in local blood flow, in the spinal cord of syrinx rats between 1 hour and 1 day time points.

### 3.3. Neuronal Mitochondrial Depletion

**Figure 1:**
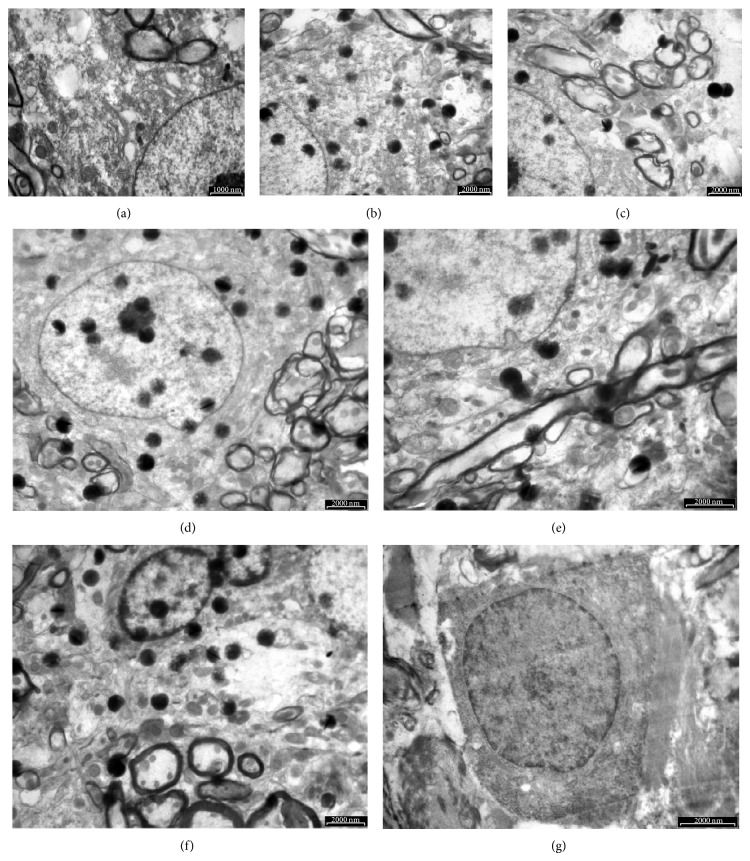
Electron micrographs of motoneuronal mitochondria in the spinal cord of intact, laminectomy and syrinx animals. Intact control (a), laminectomy at 1 h (b), 24 h (c), and 6 weeks (d) time points, syrinx animals at 1 h (e), 24 h (f), and 6 weeks (g) after syrinx induction procedure. (bar = 1,000 nm in (a), bar = 2,000 nm in (b)–(g)).

Neuronal mitochondria were observed in the grey matter of the spinal cord in the intact control (Figure 1(a)). Most of them were crista-type mitochondria. Their shape was spherical or more irregular and often bizarre with irregular crests, and even defective in the central parts. An intramitochondrial granule was seen. Neuronal mitochondria were also identified in the grey matter of the spinal cord, in the laminectomy group at all time points (Figures 1(b)–1(d)), and syrinx animals at 1 hour and 1 day time points (Figures 1(e) and 1(f)). One day after syrinx induction procedures, more “degenerated” mitochondria, were seen. These mitochondria underwent various changes displaying spherical multimembranous structures and myelin figures. At 6-week time point, swollen neurons were observed but mitochondria were completely depleted from the grey matter surrounding cyst in syrinx animals (Figure 1(g)).

**Figure 2:**
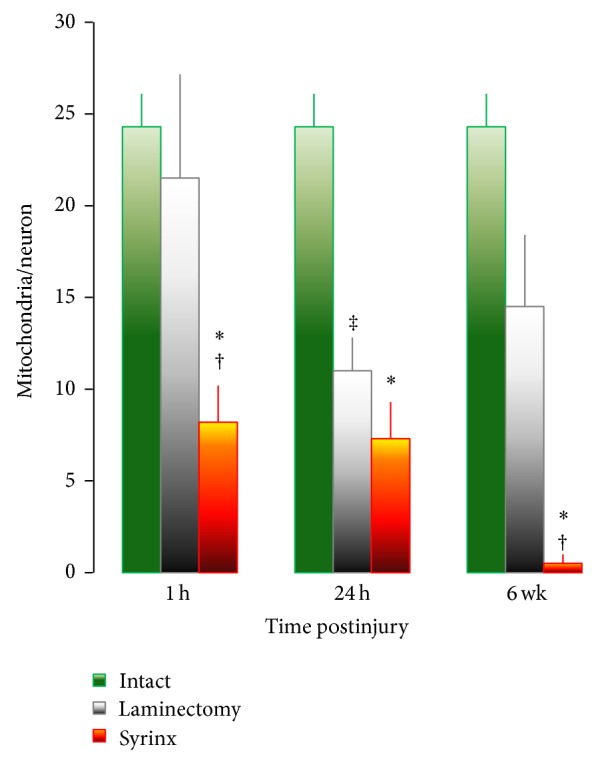
Quantification of motoneuronal mitochondria in the spinal cord of intact control, laminectomy and syrinx groups at 1 h, 24 h and 6 weeks after injury. ^*^ *P* < 0.005, versus intact control. ^†^ *P* < 0.05, versus laminectomy at the same time point. ^‡^ *P* < 0.03, versus intact control.

When evaluating the degree of degeneration, it is not the size and shape, but the number of mitochondria that is important. Quantification of neuronal mitochondria in the grey matter of the spinal cord in intact controls, laminectomy group, and syrinx animals at 1 hour, 1 day, and 6-week time points was shown in Figure 2. There was a similar number of mitochondria in intact controls and laminectomy group at 1 hour time point, the number of mitochondria declined by half in laminectomy group at 24 hours, which then recovered by 24% at 6 weeks. When compared with laminectomy, the number of mitochondria was 2.6- and 1.5-fold lower in syrinx groups at 1 hour and 1 day, respectively, and no mitochondrion was identified in the grey matter surrounding cyst at 6 weeks. Such complete depletion of mitochondria indicates dying neuron.

### 3.4. Adenine Nucleotide Changes

**Figure 3:**
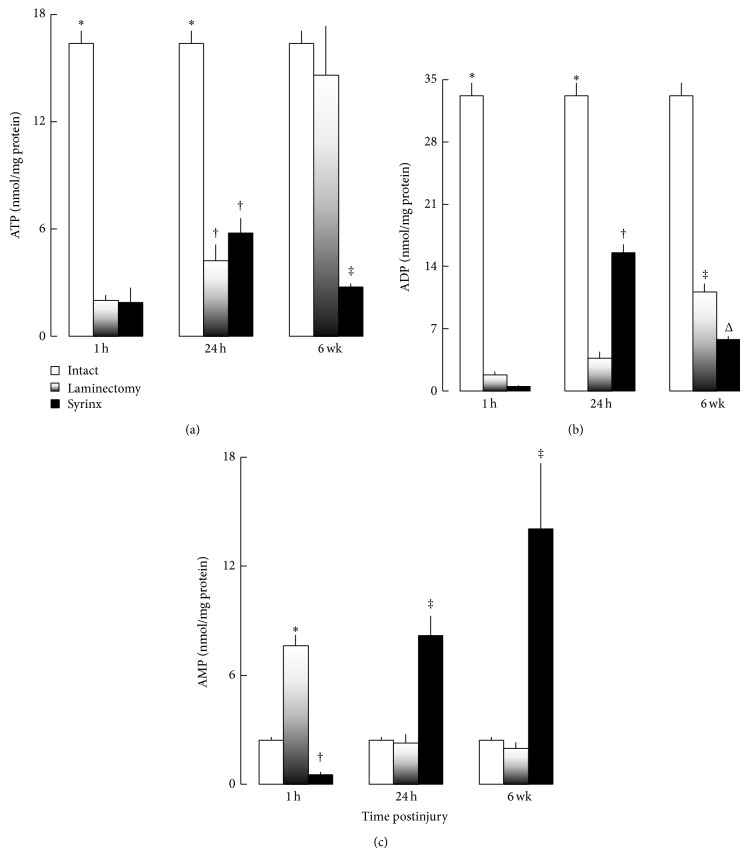
Quantification of adenine nucleotide at C8 level of the spinal cord of intact control, laminectomy and syrinx groups at 1 h, 24 h, and 6 weeks after injury. Data expressed as mean ± SE from 4–21 separate experiments. (a) ATP concentration. ^*^ *P* < 0.05, versus laminectomy and syrinx animals at the same time point. ^†^ *P* < 0.05, versus corresponding groups at 1 h and 6 weeks. ^‡^ *P* < 0.05, versus intact and laminectomy at the same time point. (b) ADP concentration. ^*^ *P* < 0.05, versus laminectomy and syrinx animals at the same time point. ^†^ *P* < 0.05, versus laminectomy at the same time point, and syrinx animals at 1 h and 6 weeks after injury. ^‡^ *P* < 0.05, versus intact, laminectomy at 1 and 24 h. ^Δ^ *P* < 0.05, versus intact and laminectomy at the same time point and syrinx group at 24 h. (c) AMP concentration. ^*^ *P* < 0.05, versus intact and syrinx at the same time point. ^†^ *P* < 0.05, versus intact and laminectomy at the same time point. ^‡^ *P* < 0.05, versus intact, laminectomy at the same time point, and syrinx at 1 h.

There is a correlation between the number of mitochondria and the energy synthesis of the cell. Adenine nucleotide levels in the spinal cord at C8 were summarized in Figure 3. Comparing with the levels of ATP, and ADP in normal controls, energy levels in the laminectomy group declined at 1 hour after injury and partially recovered at later time points. In contrast, such an initial energy reduction did not recover in the spinal cords with syringes (Figures 3(a) and 3(b)). Increasing levels of AMP with time were recorded in syrinx group (Figure 3(c)).

At 1 hour after injury, ATP content was 12% in laminectomy and syrinx of that in the intact controls (*P* < 0.01). At 1 day time point, ATP in laminectomy and syrinx was 25% of that in the intact controls (*P* < 0.05). Six weeks later, the ATP loss in laminectomy was fully re-installed but syrinx group remained at only 17% of that in the intact controls (*P* < 0.01).

ADP content in the spinal cord showed a similar pattern as ATP. At 1 hour time point, ADP content in laminectomy and syrinx animals was 5% and 2% of that in the intact controls (*P* < 0.01), respectively. At 1 day time point, ADP levels in laminectomy and syrinx rats recovered to 11% and 47% of that in the intact controls (*P* < 0.05), respectively. At 6-week time point, ADP levels in laminectomy recovered up to 34% of that in the intact controls but syrinx animals declined to 17% of that in the intact controls (*P* < 0.005).

AMP content in the spinal cord was relatively stable in the laminectomy group but showed a pattern just the opposite of ATP and ADP in syrinx group, which suggested increasing energy consumption with time after syrinx induction.

### 3.5. Neuronal Apoptosis and Necrosis

**Figure 4:**
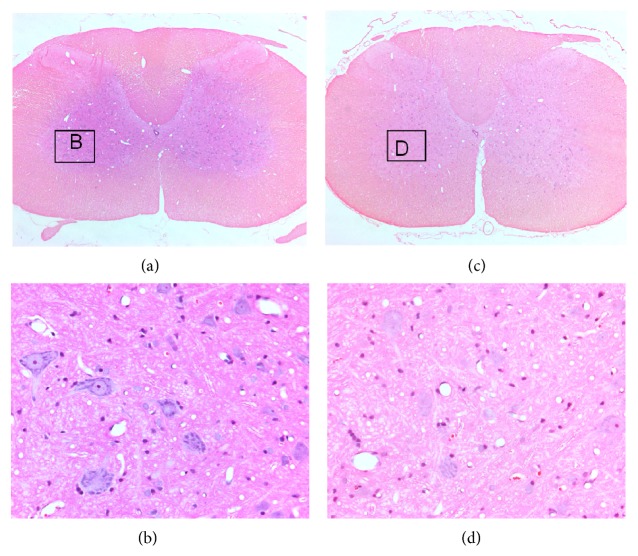
Hematoxylin and eosin (H and E) stained coronal spinal cord sections from laminectomy ((a) and (b)) and syrinx rat model of posttraumatic syringomyelia ((c) and (d)) at 1 hour after syrinx induction procedure.

To understand the consequences of ischemia in the context of apoptotic versus necrotic types of neuronal death, we evaluated morphology of apoptosis and necrosis in the spinal cord. At 1 hour time point, there was no cyst formation in the spinal cords of laminectomy or syrinx rats (Figures 4(a) and 4(c)). Ischemic changes were observed in the spinal cords of both laminectomy and syrinx rats but restricted to cells with neuronal morphology, differed in an extent between laminectomy and syrinx rats. Two distinct types of histopathological changes were observed. The first type was characteristic of apoptosis including nuclear and cytoplasmic condensation, nuclear budding, and fragmentation into membrane-bound small bodies (apoptotic bodies) (Figure 4(b)). The second type was characteristic of necrosis including pyknotic nuclei and eosinophilic, structureless cytoplasm (red neurons), or absent nuclear hematoxylin staining (ghost neurons) (Figure 4(d)).

**Figure 5:**
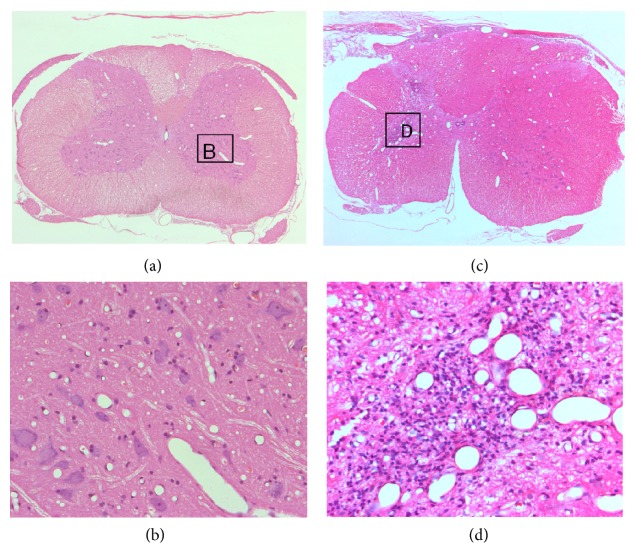
Hematoxylin and eosin (H and E) staining of coronal spinal cord sections from laminectomy ((a) and (b)) and syrinx rat model of posttraumatic syringomyelia ((c) and (d)) at 24 hours after syrinx induction procedure.

At 24-hour time point, there was no apparent cyst formation in the spinal cords of laminectomy or syrinx rats (Figures 5(a) and 5(c)). The grey matter showed ischemic cell change in the spinal cords of both laminectomy and syrinx rats. In contrast to laminectomy (Figure 5(b)), the grey matter of syrinx spinal cord appeared spongy with numerous vacuolations, and lymphocyte infiltration (Figure 5(d)).

**Figure 6:**
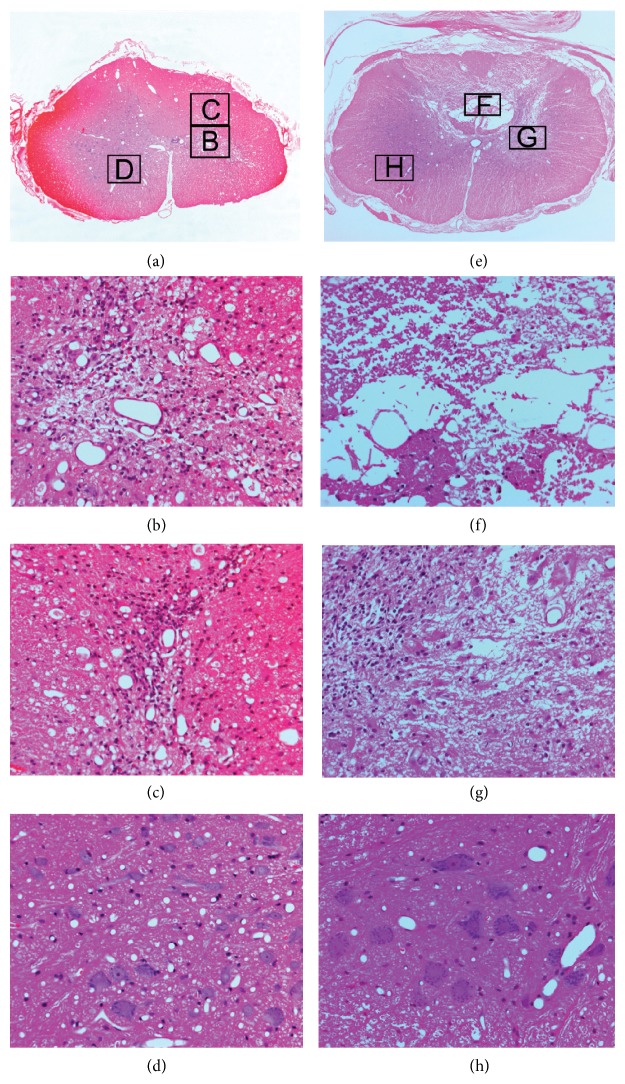
Hematoxylin and eosin (H and E) staining of coronal spinal cord sections from laminectomy ((a)–(d)) and syrinx rat model of posttraumatic syringomyelia ((e)–(h)) at 6 weeks after syrinx induction procedure.

At 6-week time point, there was no cyst formation in the spinal cords of laminectomy animals (Figures 6(a)–6(d)) although spongy with numerous vacuolations (Figures 6(b) and 6(c)), ischemic neuronal change and ghost neurons (Figure 6(d)), and lymphocyte infiltration could be seen in the grey matter. In contrast to laminectomy (Figure 6(a)), cysts were observed as cystic spaces within the cord (Figure 6(e)), sometimes containing cellular debris and a loose trabecular meshwork (Figure 6(f)) in the syrinx rats. Syringes extended from the grey matter to white matter (Figure 6(e)). The syrinx margin was defined as an interface between normal tissue and cystic spaces (Figure 6(g)). In the normal tissue, there were a small percentage of motoneurons undergoing ischemic cell change, and even ghost neurons were seen (Figure 6(h)).

**Figure 7:**
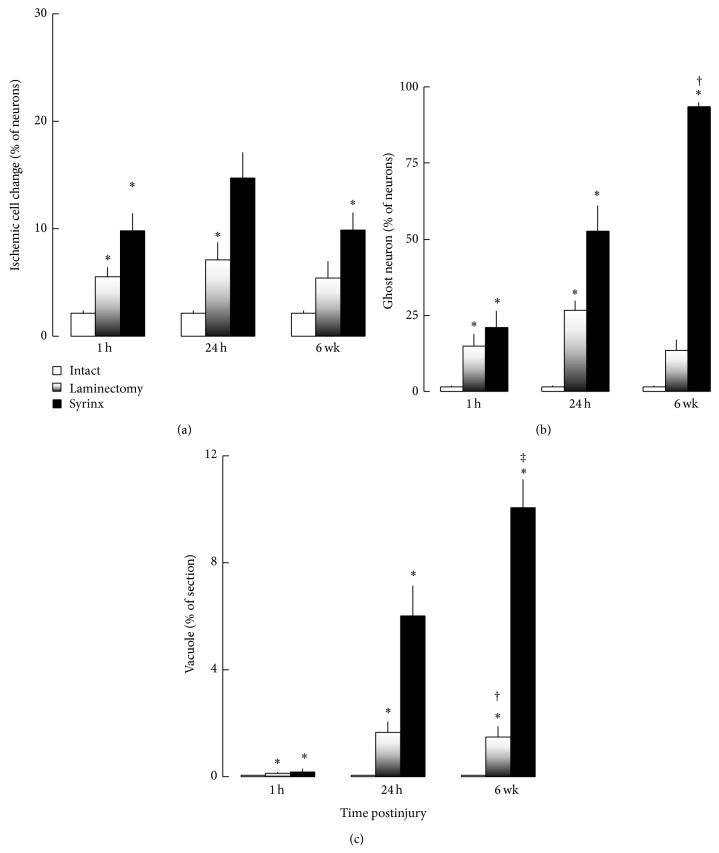
Quantification of ischemic motoneuronal change, ghost motoneuron, and spongy vacuolization in the spinal cord of intact control, laminectomy, and syrinx groups at 1 h, 24 h, and 6 weeks after injury. Data expressed as mean ± SE from 4–63 separate H and E stained sections. (a) Ischemic motoneuronal change. ^*^ *P* < 0.03, versus intact control at the same time point. Spinal cord injury, regardless of laminectomy or syrinx procedure, induced ischemic motoneuronal change. Such ischemic cell change was reversible in the spinal cord of laminectomy animals but not in syrinx rats during 6 weeks follow-up period. The initial ischemic motoneuronal change occurred in the spinal cord of syrinx rats could be recovered within 24 h following injury but it reoccurred at 6-week time point. (b) Ghost motoneurons were observed as motoneurons without nucleus. ^*^ *P* < 0.015, versus intact control at the same time point. ^†^ *P* < 0.0001, versus other groups at all 3 time points. Spinal cord injury, regardless of laminectomy or syrinx procedure, increased the percentage of ghost motoneuron. A high percentage of ghost motoneuron was reversible in the spinal cord of laminectomy animals but not in syrinx rats during 6 weeks follow-up period. There was no significant difference in the percentage of ghost motoneuron between laminectomy groups. At 6-week-time point, the percentage of ghost motoneuron was greater in syrinx than any other groups at every time point. (c) The percentage of vacuoles in a coronal spinal cord section. ^*^ *P* < 0.05, versus intact control at the same time point. ^†^ *P* < 0.02, versus laminectomy at 1 h and syrinx at 1 h. ^‡^ *P* < 0.01, versus other groups at all 3 time points except syrinx at 24 h. Spinal cord injury, regardless laminectomy or syrinx procedure, increased the percentage of vacuoles in the spinal cord. A higher percentage of vacuoles was observed at 6 h in the laminectomy group than at 1 h in both laminectomy and syrinx animals. At 6-week time point, the percentage of ghost motoneuron was greater in syrinx than any other groups at every time point except syrinx at 24 h.

The number of motoneurons with the degenerative morphologies was summarized in Figure 7. There were 2% motoneurons with ischemic cell changes per spinal cord cross section in intact control (Figure 7(a)). In the laminectomy group, this percentage increased by 2.6- and 3.3-fold at 1 hour and 24 hours, respectively (*P* < 0.03), and partially recovered by 24% 6 weeks later. In syrinx group, the percentage of ischemic cell changes increased by 4.6- and 6.9-fold at 1 hour and 24 hours, respectively (*P* < 0.02), and sustained at 1 hour level 6 weeks later. In addition, there were 1% motoneurons being identified as ghost neuron per spinal cord cross section in the intact controls (Figure 7(b)). Such a percentage increased significantly by 10.4- and 18.6-fold at 1 hour and 24 hours, respectively (*P* < 0.02) before a partial recovery from injury to 1 hour level 6 weeks later. In contrast, the percentage of ghost neuron linearly increased with time by 14.7-, 36.7-, and 65.3-fold at 1 hour, 24 hours, and 6 weeks in syrinx group.

Heterogeneous vacuolization of membranous organelles such as mitochondria within motoneurons was observed in the spinal cord after syrinx induction procedure. There was no such vacuole identified in the intact controls, and only a negligible number of vacuole was observed in laminectomy and syrinx at 1 hour time point. The percentage of vacuole area per spinal cord cross section became obvious at 24 hours onwards in laminectomy and syrinx groups. There was 1-2% of vacuole area per section in the spinal cord of laminectomy animals at 24 hours and 6 weeks (Figure 7(c)). This percentage increased by 3.6- and 6.1-fold in syrinx at 24 hours and 6 weeks, respectively.

**Figure 8:**
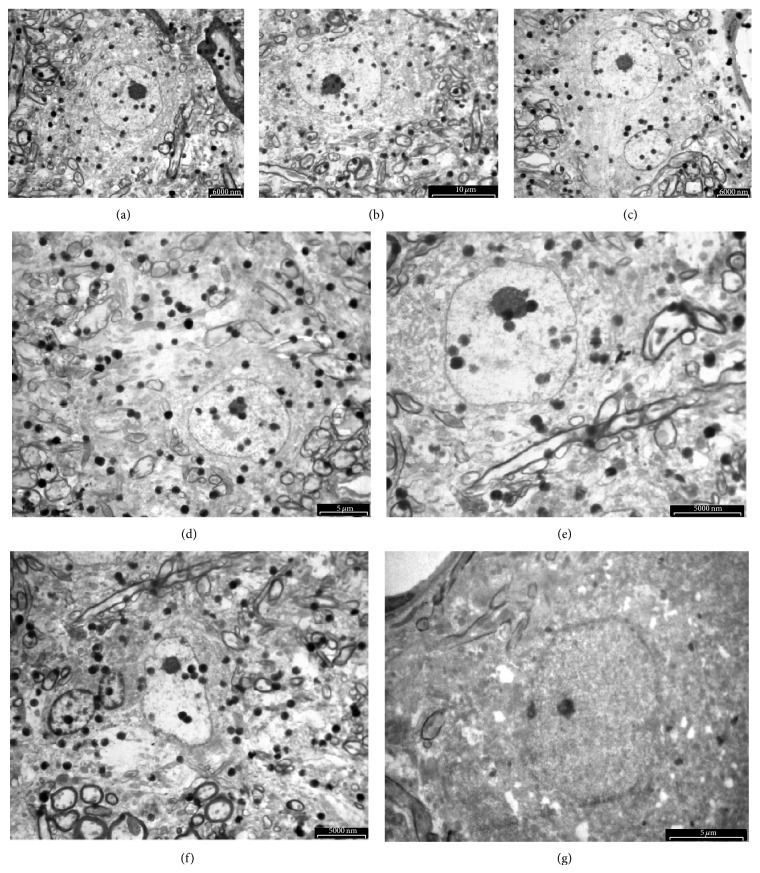
Ultrastructural time course of cellular changes associated with quisqualic acid excitotoxicity in the motoneuron of the spinal cord: NMDA receptor-mediated excitotoxicity resembles necrosis, and cellular changes induced by laminectomy in the motoneuron of the spinal cord: apoptosis. Electron micrographs illustrate motoneuronal changes associated with the necrotic-like degenerative neuronal morphology induced by quisqualic acid ((c), (e), (g)). A normal motoneuron from the spinal cord is shown for comparison in (a). Selected motoneurons displaying the predominant morphological changes at the different times after quisqualic acid injection (1 h, 24 h, and 6 weeks) were arranged in a temporal sequence to show the progression of excitotoxic injury ((c), (e), (g)), apoptotic changes in the laminectomy group were shown at corresponding time points ((b), (d), (f)). Refer to Section 3 for a description of nuclear changes.

Ultrastructural changes associated with neuronal apoptosis and necrosis in the spinal cord of normal, laminectomy, and quisqualic acid injected were shown in Figure 8. The definitive changes to distinguish apoptosis and necrosis classically occur within the nucleus. Comparing with a normal control neuron (Figure 8(a)), the size of a nucleus was essentially the same at 1 hour, 24 hours, and 6 weeks of laminectomy (Figures 8(b), 8(d) and 8(f)) and quisqualic acid injected spinal cord (Figures 8(c), 8(e), and 8(g)). There was a round nucleus at an early stage of the process towards spindle-shape at 1 hour of laminectomy (Figure 8(b)). Neurons in 1 hour of quisqualic acid injected spinal cord showed essentially the same size of a round nucleus as the normal control neuron (Figure 8(c)).

## 4. Discussion

Previous reports have unveiled that combining an excitotoxic insult with kaolin-induced arachnoiditis produces syrinx in the grey matter of the spinal cord, which mimics the human disease [12, 13]. This study provides the evidence for the local ischemia correlated mitochondrial dysfunction of neuron cells during the pathological process after traumatic injury to the spinal cord. In accordance with previous reports [12, 13, 18, 19], the size of enlarged syringes was 15% of the cross-sectional area in the grey matter at the C8 level and could extend up to 3 segments in length by 6 weeks after syrinx induction procedure (Figure 1). The cavities are fluid filled. It is possible that the imbalance of CSF inflow and outflow generates intrasyringal pressure against syrinx walls causing compression. As a consequence, local blood flow declined by 86%. This pressure-induced regional ischemia surrounding the cyst is likely responsible for irreversible mitochondrial permeability transition.

The mitochondrial permeability transition occurs by opening of the permeability transition pore, which conducts freely solutes of molecular weight ≤1.5 kDa [25]. As a consequence, mitochondria depolarize, uncouple, and undergo large amplitude swelling. Mitochondria are recognized as target organelles for the regulation and execution of neuronal death under pathological conditions [10]. Transmission electron microscopy reveals the swelling of mitochondria in the process of glutamate neurotoxicity shortly after microinjection of quisqualic acid (Figure 2(e)). There are four mechanisms of neurotoxicity related to mitochondria [26]. First, the entry and accumulation of cytoplasmic Ca^2+^ through ionotropic glutamate receptors results in the subsequent accumulation of Ca^2+^ in neuronal mitochondria ([Ca^2+^]_m_) [27, 28]. The cytoplasmic Ca^2+^ is transported into mitochondria through an electrophoretic uniporter. Its driving force is generated by the negative membrane potential, ΔΨ_m_ [29]. Second, neuronal mitochondria become depolarized, due to the transport of Ca^2+^ into the matrix and the inhibition of the oxidative phosphorylation [30, 31]. Third, inhibiting the mitochondrial Ca^2+^ uptake reduces the Ca^2+^-mediated glutamate neurotoxicity [32]. Finally, increasing the mitochondrial membrane and redox potential blocks the accumulation in [Ca^2+^]_i_ and neuronal death following the activation of *N*-methyl-D-aspartate (NMDA) receptors [33]. Excess Ca^2+^ in mitochondria results in mitochondrial dysfunction and neuronal death. With the accumulation of [Ca^2+^]_m_, ATP synthesis in mitochondria is impaired, due to the collapse of the mitochondrial oxidative phosphorylation [34, 35]. ATP depletion will interfere with the actions of ATP-dependent Ca^2+^ pumps, amplify the accumulation of [Ca^2+^]_i_, therefore, enhancing the process of glutamate neurotoxicity resulting in neuronal death. The activation of NMDA receptors by quisqualic acid produces reactive oxygen species in mitochondria, primarily through the inhibition of the oxidative phosphorylation and membrane potential [31, 33]. Ca^2+^-induced mitochondrial damage causes increased mitochondrial membrane permeability [36], which can result in the mitochondrial release of cytotoxic substances.

Neurons are normally exposed to minimum levels of free radicals from exogenous and endogenous sources. Since neurons have a minimum storage capacity for oxygen and a high probability of lipid peroxidation, they are especially vulnerable to free radical-medicated injury. Hasegawa et al. [37] provided evidence that neuronal mitochondria are free radical sources during ischemia. Mitochondria produce ATP by utilizing the majority of O_2_ (about 90%), which is taken up by neurons. During electron transfer in the inner mitochondrial membrane, electrons spontaneously leak from the electron transport chain and react with the available O_2_ to produce superoxide. The superoxide is normally cleared to H_2_O by superoxide dismutases and glutathione peroxidase. During ischemia, however, many free radicals cannot be cleared by antioxidant enzymes [38]. Calcium accumulates in the cytosol and enters the mitochondria, which results in the production of mitochondrial free radicals [39–42]. Excess Ca^2+^ in the mitochondria interrupts the electron transport chain and collapses the neuronal mitochondrial membrane potential [43]. Therefore, free electrons are accumulated in the mitochondria, which react with oxygen and cause the production of superoxide. The superoxide is further processed to produce the hydroxyl radical by a Fenton reaction or peroxynitrite by reacting with nitric oxide. The reactive oxygen and nitrogen species also inhibit the electron transport chain in the mitochondria and amplify a generation of mitochondrial free radicals [43]. Thus, mitochondrial oxidative damage involves mitochondrial lipid peroxidation [44] and mitochondrial DNA oxidation [41]. Free radical neurotoxicity demonstrated the occurrence of typical necrosis in neurons, which is evident by the swelling of mitochondria and neuronal cell body, scattering condensation of nuclear chromatin, and fenestration of the nuclear membrane prior to complete depletion of neuronal mitochondria lately in the process of glutamate neurotoxicity (Figures 2(f) and 3).

The decrease of local blood flow also induces necrapoptosis by the accumulation of lactate and decline in ATP level (Figure 4(a)) [45]. The intracellular acidification by excess CO_2_, and the dysfunction of active ion pumps by loss of ATP is known as a primary cause of neuronal necrosis [46]. Several lines of evidence suggest that the intracellular acidification comprises an event in the process of apoptosis as well, such as an increase of interleukin-1*β* converting enzyme (ICE)-like protease activity [47, 48]. The collapse of the proton gradient across the mitochondrial inner membrane by a decline of ATP can result in a complete loss of the mitochondrial membrane potential. The cytochrome *c* in the cytosol that is released by the permeability of the out mitochondrial membrane binds to apoptotic protease activating factor 1, activates procaspase-9, and leads to the activation of downstream caspases, such as caspase-3 [49]. The intracellular acidification causes apoptosis in cultured neurons [50]. It is possible that ischemia-induced acidosis may mediate neuronal necrapoptosis.

Arachnoid adhesion may also affect spinal cord blood flow. However, clinical studies demonstrate a rapid increase in spinal cord blood flow following decompression of an ischemic spinal cord, suggesting that arachnoid adhesion-induced ischemia is a minor component of the predecompression reduction in blood flow [15, 16, 51].

There is a negative correlation between the severity of spinal cord injury and the degree of acute ischemia at the side of injury [52–57]. Immediately following milder spinal injury, there may be no measurable change of spinal cord blood flow from baseline values [58, 59]. The lack of a reduction in local blood flow in the spinal cord between 1 hour and 1 day after syrinx induction as well as the preservation of function in laminectomy following injury, suggests that mechanical injury of our syrinx induction procedures can be viewed as a mild form of trauma [12]. Shock wave occurred following the mechanical injury of spinal cord alters the mitochondrial electron transport-oxidative phosphorylation sequence resulting in significant reductions, in the rate of mitochondrial respiration and depression of energy metabolism (Figure 4). Cessation of mitochondrial ATP production by injury stress can be partially restored by anaerobic glycolysis since neurons possess both glucokinase and hexokinase [60–63], which is in an attempt to reverse neuronal necrapoptosis or convert necrosis to apoptosis [64]. Preservation of cell viability is evidenced by full replacement of ATP level and partial recovery of neuronal mitochondria in the laminectomy group at 6-week time point (Figures 3 and 4).

Spinal cord injury consists in part of mechanical disruption of neuronal and vascular tissue and much of it could be irreversible. The damage can be simplified as two processes: the initial traumatic disruption of tissue from direct impact of shock waves and the secondary processes set in response to the primary insult. The secondary processes, including ischemia induced mitochondrial permeability transition, could start immediately at the time of insult and are not clinically apparent for hours to days, which may provide a “window of opportunity” for treatment of the developing neurotoxicity. They comprise a complex series of pathophysiological events leading to necrapoptosis of surrounding tissue originally spared from the primary injury. For example, the mitochondrial permeability transition could be inhibited pharmacologically, with the immunosuppressant, cyclosporine-A [65]. Thus, the therapeutic intervention of ischemic neurotoxicity should be aimed to early prevent glutamate neurotoxicity, oxidative stress, and apoptosis or necrapoptosis.

## 5. Conclusions

Combining an excitotoxic insult with kaolin-induced arachnoiditis causes local ischemia in the spinal cord and subsequent onset of neuronal mitochondrial depletion and profound ATP loss. These consequences of glutamate neurotoxicity and neuron cell necrosis play an important role in the cavity enlargement of posttraumatic syringomyelia. In contrast, ischemia does not occur following mechanical injury, such as laminectomy, in which mitochondrial loss and decline in ATP are reversible. Recovery of neuronal mitochondria and ATP may reverse neuron cell death pathways. This explains the observation why no syrinx forms after laminectomy.

## Abbreviations

ADP: Adenosine diphosphate
AMP: Adenosine monophosphate
AMPA: *α*-amino-3-hydroxy-5-methyl-4-isoxazolepropionic acid
ATP: Adenosine triphosphate
CSF: Cerebrospinal fluid
EDTA: Ethylene dinitrilotetra-acetic acid
EtOH: Ethanol
H&E: Hematoxylin and eosin
ICE: Interleukin-1*β* converting enzyme
NMDA: *N*-methyl-D-aspartate
NMDARs: *N*-methyl-D-aspartate receptors
SPSS: Statistical package for the social sciences.
